# High glycated albumin is associated with early neurological deterioration in patients with acute ischemic stroke

**DOI:** 10.1186/s12883-024-03747-4

**Published:** 2024-08-10

**Authors:** Ki-Woong Nam, Jung Hoon Han, Chi Kyung Kim, Hyung-Min Kwon, Yong-Seok Lee, Kyungmi Oh, Keon-Joo Lee, Byeongsu Park

**Affiliations:** 1 Department of Neurology, Seoul Metropolitan Government-Seoul National University Boramae Medical Center, Seoul, Korea; 2 Department of Neurology, Seoul National University College of Medicine, Seoul, Korea; 3 Department of Neurology, Korea University Guro Hospital, 148 Gurodong-ro, Guro-gu, Seoul, 08308 Korea; 4 Department of Neurology, Korea University College of Medicine, Seoul, Korea; 5 Department of Radiology, Korea University Guro Hospital, Seoul, Korea

**Keywords:** Ischemic stroke, Glycated albumin, Glucose, Prognosis, Early outcome

## Abstract

**Background:**

Glycated albumin (GA) is an indicator of glycemic variability over the past 2–4 weeks and has suitable characteristics for predicting the prognosis of ischemic stroke during the acute phase. This study evaluated the association between early neurological deterioration (END) and GA values in patients with acute ischemic stroke (AIS).

**Methods:**

We assessed consecutive patients with AIS between 2022 and 2023 at two large medical centers in Korea. END was defined as an increase of ≥ 2 in the total National Institutes of Health Stroke Scale (NIHSS) score or ≥ 1 in the motor NIHSS score within the first 72 h of admission. We evaluated various glycemic parameters including fasting glucose (mg/dL), hemoglobin A1c (%), and GA (%).

**Results:**

In total, 531 patients with AIS were evaluated (median age: 69 years, male sex: 66.3%). In the multivariable logistic regression analysis, GA value was positively associated with END (adjusted odds ratio [aOR] = 3.24, 95% confidence interval [CI]: 1.10–9.50). Initial NIHSS score (aOR = 1.04, 95% CI: 1.01–1.08) and thrombolytic therapy (aOR = 2.06, 95% CI: 1.14–3.73) were also associated with END. In a comparison of the predictive power of glycemic parameters for END, GA showed a higher area under the curve value on the receiver operating characteristic curve than fasting glucose and hemoglobin A1c.

**Conclusions:**

High GA values were associated with END in patients with AIS. Furthermore, GA was a better predictor of END than fasting glucose or hemoglobin A1c.

**Supplementary Information:**

The online version contains supplementary material available at 10.1186/s12883-024-03747-4.

## Background

Diabetes and hyperglycemia are well-known risk factors for ischemic stroke [1]. Elevated blood glucose levels not only increase the risk of ischemic stroke but also have a significant impact on both short- and long-term outcomes [2–4]. European and US guidelines recommend glucose control within appropriate levels in patients with ischemic stroke and suggest plasma glucose and hemoglobin A1c as monitoring indicators for this purpose [5–7]. However, plasma glucose is greatly influenced by diet, type and timing of glucose-lowering agents, and accompanying medical comorbidities. In addition, it can temporarily increase as an acute-phase reactant after a stroke [8, 9]. In contrast, hemoglobin A1c is a relatively stable reflector of glycemic status [10]. However, since hemoglobin A1c reflects data from the past 2–3 months, its usefulness as an indicator of prognosis or treatment response during the acute phase of ischemic stroke is limited.

The glycated albumin (GA) level is an indicator of short-term glycemic variability [11]. GA reflects the glycemic variability over the past 2–4 weeks due to the rapid turnover of albumin and its high glycation rate [12–14]. Recently, glycemic variability has been recognized to play an important role in the development of microvascular- and macrovascular complications in diabetic patients, as much as chronic hyperglycemia [11, 15]. Therefore, GA is considered a useful indicator of glycemic control. Furthermore, GA has the advantage that it is not overly affected by external factors as much as plasma glucose and can be used even in medical conditions where the value of hemoglobin A1c is difficult to reliably assess (e.g., anemia, severe kidney disease, hemoglobinopathy) [14, 16]. GA has been used in several studies and is associated with various diseases, including atherosclerosis, peripheral artery disease, and cardiovascular and cerebrovascular diseases [10, 14, 16–18]. 

Considering its reflection period (past 2–4 weeks), GA appears to be suitable for reflecting glycemic variability prior to the onset of ischemic stroke as well as glycemic status and treatment effects during acute periods [11]. In fact, GA values are associated with various short- and long-term outcomes in patients with ischemic stroke [11, 12, 19–21]. A previous study examined the association between GA and early neurological deterioration (END) in patients with acute ischemic stroke (AIS) [15]. However, this study only dealt with patients with AIS and prediabetes, and there were limitations due to the small sample size and END events.

In this study, we evaluated the association between GA values and END in patients with AIS, based on data from two large medical centers in Korea. In addition, by comparing the predictive power of GA and other glycemic parameters for END, we investigated which glycemic parameter was most suitable for predicting acute outcomes in patients with stroke.

## Methods

### Study population

From the consecutive stroke registries of two large medical centers in Korea (Seoul Metropolitan Government-Seoul National University Boramae Medical Center and Korea University Guro Hospital), we included patients diagnosed with AIS between June 2022 and February 2023. Starting in 2022, these two centers measured GA levels in patients with AIS as part of their risk factor assessment. In addition, all AIS patients underwent a broad etiological evaluation, including brain magnetic resonance imaging, magnetic resonance angiography, echocardiography, and laboratory examinations. According to the exclusion criteria, patients who met the following criteria were excluded from the analysis: (1) arrival > 72 h after symptom onset, (2) patients without GA data. Finally, 531 patients with AIS were included in the final analysis.

### Demographic, clinical, and laboratory variables

Baseline demographic and clinical variables were evaluated, including age, sex, hypertension, diabetes, dyslipidemia, atrial fibrillation, ischemic heart disease, chronic kidney disease, current smoking status, initial stroke severity, stroke mechanism, and use of thrombolytic therapy. The initial stroke severity was assessed daily from admission to discharge by a skilled neurologist not involved in the study using the National Institutes of Health Stroke Scale (NIHSS) score. The stroke mechanism was classified according to the Trial of Org 10,172 in Acute Stroke Treatment (TOAST) classification based on the evaluation results until discharge [22]. Thrombolytic therapy included intravenous thrombolysis and intra-arterial thrombectomy.

Laboratory examinations were performed within the first 24 h of admission. This included fasting glucose (mg/dL), hemoglobin A1c (%), GA (%), low-density lipoprotein (LDL) and high-density lipoprotein (HDL) cholesterol (mg/dL), triglycerides (mg/dL), white blood cell (WBC) counts (x 10^3^/uL), and high-sensitivity C-reactive protein (hs-CRP, mg/dL). GA was obtained from the venous blood collected after a minimum of 8 h of fasting. The collected serum samples were analyzed using an enzymatic method employing albumin-specific proteinase and ketoamine oxidase. In our center, the normal range of GA values is between 11.0% and 16.0%.

### Outcome variables

As the main outcome variable of this study, END was defined as an increase of two or more points in the total NIHSS score or an increase of one or more points in the motor NIHSS score within the first 72 h after admission, as in previous studies [23]. In addition, we used the modified Rankin Scale (mRS) score to measure the functional outcomes of the patients at the time of discharge. We defined an unfavorable outcome as discharge with an mRS score ≥ 3 [24]. 

### Statistical analysis

Continuous variables with normal distributions are presented as mean ± standard deviation, whereas the others are presented as median + interquartile ranges. As GA may be an unfamiliar parameter, we analyzed the relationship between GA and various demographic, clinical, and laboratory variables to identify the characteristics of patients with high GA levels. Simple linear regression analysis was used for these analyses. Continuous variables with skewed data were adjusted by using logarithmic scales.

Subsequently, we performed a univariate analysis to identify the parameters associated with END occurrence. We used the Student’s *t*-test or Mann-Whitney *U*-test for continuous variables and the chi-squared test or Fisher’s exact test for categorical variables. Based on the results of the univariate analysis, variables with *P* < 0.05 and age were introduced as confounders in the multivariable logistic regression analysis. Considering the potential interaction and multicollinearity, fasting glucose and hemoglobin A1c levels were not included in the multivariable analysis, along with GA. In addition, we used GA not only as a continuous variable, but also as a categorical variable based on an appropriate cut-off value in the analysis. Based on previous studies, “GA > 16.0%” was used as the cut-off value [11, 19]. 

As ischemic stroke has heterogeneous mechanisms, we also compared the impact of GA on END according to the stroke mechanism. In addition, we compared the predictive power of various glycemic parameters for END occurrence. We drew receiver operating characteristic (ROC) curves and calculated the area under the curve (AUC) to quantify the predictive power. In addition, we compared the adjusted odds ratios (aORs) of each glycemic parameter based on their well-established cut-off values in the multivariable logistic regression analysis. All the statistical analyses were performed using SPSS version 23.0 (IBM Corp., Armonk, NY, USA). All variables with *P* < 0.05 were considered statistically significant.

## Results

In total, 531 patients with AIS were evaluated (median age: 69 years, male sex: 66.3%). The median initial NIHSS score was 3 [2–7], and the median value of GA was 14.7 [13.1–17.0] %. END occurred in 96 (18.1%) patients. Other baseline characteristics are listed in Table 1.

**Table 1:** Baseline characteristics of the study population (*n* = 531)

**Demographic & clinical factors**	
Age, y [IQR]	69 [60–78]
Sex, male, n (%)	352 (66.3)
Visit time, h [IQR]	6.1 [1.8–20.8]
Hypertension, n (%)	360 (67.8)
Diabetes, n (%)	196 (36.9)
Dyslipidemia, n (%)	278 (52.4)
Atrial fibrillation, n (%)	88 (16.6)
Ischemic heart disease, n (%)	57 (10.7)
Chronic kidney disease, n (%)	54 (10.2)
Current smoking, n (%)	138 (26.0)
Initial NIHSS score, [IQR]	3 [2-7]
Stroke mechanism, n (%)	
Large artery atherosclerosis	154 (29.0)
Small vessel occlusion	140 (26.4)
Cardioembolism	116 (21.8)
Other determined	13 (2.4)
Undetermined	108 (20.3)
Thrombolytic therapy, n (%)	76 (14.3)
**Laboratory factors**	
Hemoglobin A1c, % [IQR]	5.9 [5.6–6.6]
Fasting glucose, mg/dL [IQR]	117 [98–153]
Glycated albumin, % [IQR]	14.7 [13.1–17.0]
LDL cholesterol, mg/dL [IQR]	97 [73–126]
HDL cholesterol, mg/dL [IQR]	43 [37–51]
Triglyceride, mg/dL [IQR]	100 [73–145]
White blood cell counts, x 10^3^/uL [IQR]	7.5 [6.1–9.4]
High-sensitivity C-reactive protein, mg/dL [IQR]	0.98 [0.32–2.57]
**Outcome variables**	
Early neurological deterioration, n (%)	96 (18.1)
Discharge mRS score ≥ 3	211 (39.7)

NIHSS = National Institutes of Health Stroke Scale, LDL = low-density lipoprotein, HDL = high-density lipoprotein, mRS = modified Rankin Scale

In our study population, GA levels were positively correlated with age, hypertension, diabetes, dyslipidemia, chronic kidney disease, hemoglobin A1c, fasting glucose levels, and hs-CRP levels. In contrast, GA levels were negatively correlated with HDL cholesterol levels (Table 2).

**Table 2:** Simple linear regression analysis between glycated albumin^*^ and demographic, clinical, and laboratory risk factors

	β (95% CI)	*P* value
Age	0.004 (0.002 to 0.005)	< 0.001
Male sex	-0.023 (-0.066 to 0.019)	0.288
Hypertension	0.060 (0.017 to 0.103)	0.006
Diabetes	0.261 (0.226 to 0.296)	< 0.001
Dyslipidemia	0.051 (0.011 to 0.091)	0.013
Atrial fibrillation	-0.003 (-0.057 to 0.051)	0.917
Ischemic heart disease	0.005 (-0.060 to 0.070)	0.891
Chronic kidney disease	0.126 (0.060 to 0.192)	< 0.001
Current smoking	-0.024 (-0.069 to 0.022)	0.311
Initial NIHSS score, [IQR]	0.001 (-0.002 to 0.005)	0.398
Stroke mechanism, n (%)		
Large artery atherosclerosis	0.033 (-0.011 to 0.077)	0.143
Small vessel occlusion	-0.037 (-0.083 to 0.009)	0.112
Cardioembolism	-0.023 (-0.071 to 0.026)	0.360
Other determined	-0.041 (-0.171 to 0.089)	0.537
Undetermined	0.032 (-0.018 to 0.082)	0.206
Thrombolytic therapy, n (%)	-0.009 (-0.067 to 0.048)	0.751
Hemoglobin A1c, % [IQR] ^*^	0.972 (0.900 to 1.043)	< 0.001
Fasting glucose, mg/dL [IQR] ^*^	0.392 (0.345 to 0.440)	< 0.001
LDL cholesterol, mg/dL [IQR]	0.000 (-0.001 to 0.001)	0.938
HDL cholesterol, mg/dL [IQR] ^*^	-0.094 (-0.175 to -0.012)	0.024
Triglyceride, mg/dL [IQR] ^*^	0.010 (-0.030 to 0.050)	0.629
White blood cell counts, x 10^3^/uL [IQR] ^*^	0.022 (-0.038 to 0.082)	0.469
Hs-CRP, mg/dL [IQR] ^*^	0.015 (0.003 to 0.026)	0.014

NIHSS = National Institutes of Health Stroke Scale, LDL = low-density lipoprotein, HDL = high-density lipoprotein, hs-CRP = high-sensitivity C-reactive protein

^*^These variables were log-transformed

Compared with the non-END group, the END group had a higher frequency of diabetes and thrombolytic therapy, as well as higher initial NIHSS scores, hemoglobin A1c levels, fasting glucose levels, GA levels, and WBC counts (Table 3). Multivariable logistic regression analysis demonstrated that higher GA levels were closely associated with END, even after adjusting for confounding factors (adjusted odds ratio [aOR] = 3.24, 95% confidence interval [CI]: 1.10–9.50). Initial NIHSS score (aOR = 1.04, 95% CI: 1.01–1.08) and thrombolytic therapy (aOR = 2.06, 95% CI: 1.14–3.73) were also positively associated with END, being independent from glycated albumin levels. When the multivariable analysis was performed on the basis of the cut-off point, “GA > 16.0%” also showed a close statistical association with END (aOR = 1.82, 95% CI: 1.05–3.15; Table 4).

**Table 3:** Baseline characteristics of groups with and without early neurological deterioration

	Non-END (*n* = 435)	END (*n* = 96)	*P*-value
Age, years [IQR]	69 [59–78]	72 [62–80]	0.079
Sex, male, n (%)	293 (67.4)	59 (61.5)	0.269
Visit time, h [IQR]	6.5 [1.9–20.8]	5.0 [1.4–22.2]	0.534
Hypertension, n (%)	291 (66.9)	69 (71.9)	0.345
Diabetes, n (%)	152 (34.9)	44 (45.8)	0.045
Dyslipidemia, n (%)	224 (51.5)	54 (56.3)	0.398
Atrial fibrillation type, n (%)	75 (17.2)	13 (13.5)	0.378
Ischemic heart disease, n (%)	45 (10.3)	12 (12.5)	0.537
Chronic kidney disease, n (%)	41 (9.4)	13 (13.5)	0.227
Current smoking, (%)	113 (26.0)	25 (26.0)	0.990
Initial NIHSS score, [IQR]	3 (1–6)	5 (3–11)	< 0.001
Stroke mechanism, n (%)			0.548
Large artery atherosclerosis	126 (29.0)	28 (29.2)	
Small vessel occlusion	121 (27.8)	19 (19.8)	
Cardioembolism	92 (21.1)	24 (25.0)	
Other determined	10 (2.3)	3 (3.1)	
Undetermined	86 (19.8)	22 (22.9)	
Thrombolytic therapy, n (%)	51 (11.7)	25 (26.0)	< 0.001
Hemoglobin A1c, % [IQR]	5.9 [5.5–6.5]	6.2 [5.6–7.4]	0.016
Fasting glucose, mg/dL [IQR]	113 [98–149]	125 [105–716]	0.013
Glycated albumin, % [IQR]	14.5 [13.0-16.6]	15.8 [14.3–18.8]	< 0.001
LDL cholesterol, mg/dL [IQR]	96 [73–124]	102 [73–137]	0.316
HDL cholesterol, mg/dL [IQR]	43 [37–51]	44 [36–51]	0.709
Triglyceride, mg/dL [IQR]	100 [75–142]	100 [68–148]	0.516
White blood cell counts, x 10^3^/uL [IQR]	7.4 [6.0-9.3]	8.0 [6.6–9.7]	0.030
Hs-CRP, mg/dL [IQR]	1.03 [0.32–2.52]	0.70 [0.26–2.80]	0.612

END = early neurological deterioration, NIHSS = National Institutes of Health Stroke Scale, LDL = low-density lipoprotein, HDL = high-density lipoprotein, hs-CRP = high-sensitivity C-reactive protein

**Table 4:** Multivariable logistic regression analysis of possible predictors of early neurological deterioration

	Crude OR (95% CI)	*P*-value	Adjusted OR (95% CI)	*P*-value
**Model 1 (continuous)**				
Age	1.02 [1.00-1.03]	0.094	1.01 [0.99–1.03]	0.202
Diabetes	1.58 [1.01–2.46]	0.046	1.12 [0.64–1.95]	0.692
Initial NIHSS score	1.06 [1.03–1.09]	< 0.001	1.04 [1.01–1.08]	0.021
Thrombolytic therapy	2.65 [1.54–4.56]	< 0.001	2.06 [1.14–3.73]	0.017
Glycated albumin^*^	3.86 [1.63–9.14]	0.002	3.24 [1.10–9.50]	0.033
WBC counts^*^	1.97 [1.02–3.82]	0.045	1.69 [0.85–3.37]	0.135
**Model 2 (Categorical)**				
Age	1.02 [1.00-1.03]	0.094	1.01 [0.99–1.03]	0.256
Diabetes	1.58 [1.01–2.46]	0.046	1.15 [0.67–1.97]	0.621
Initial NIHSS score	1.06 [1.03–1.09]	< 0.001	1.04 [1.01–1.08]	0.021
Thrombolytic therapy	2.65 [1.54–4.56]	< 0.001	2.10 [1.16–3.80]	0.015
GA > 16.0%	2.09 [1.34–3.27]	0.001	1.82 [1.05–3.15]	0.034
WBC counts^*^	1.97 [1.02–3.82]	0.045	1.77 [0.89–3.53]	0.103

NIHSS = National Institutes of Health Stroke Scale, WBC = white blood cell

^*^These variables were log-transformed

**Fig. 1:**
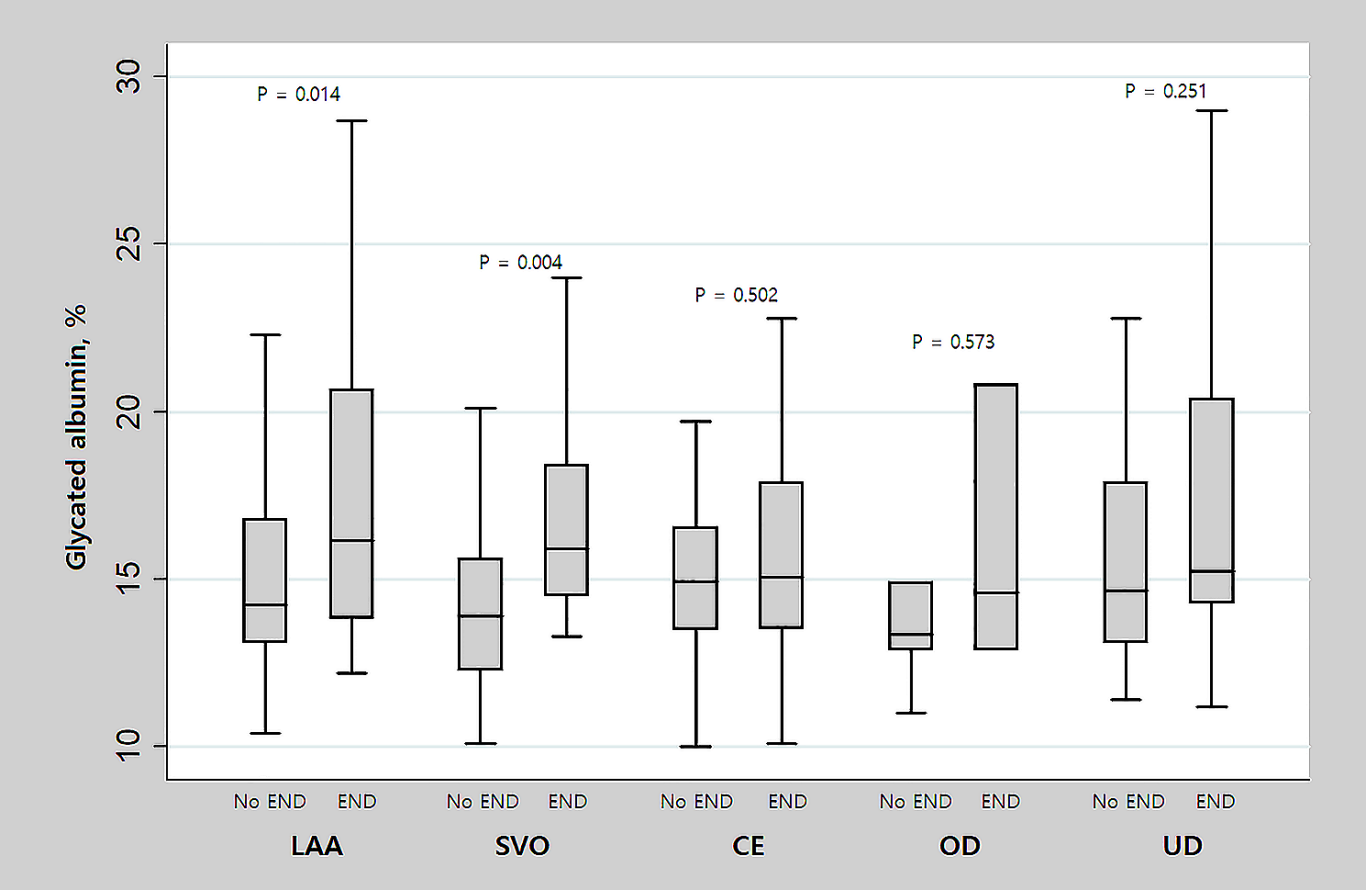
Comparison of glycated albumin levels between END and non-END groups according to stroke mechanisms. END = early neurological deterioration, LAA = large artery atherosclerosis, SVO = small vessel occlusion, CE = cardioembolism, OD = other determined, UD = undetermined. Among various mechanisms of stroke, statistically significant differences in glycated albumin levels were observed between the END and non-END groups only in stroke patients with LAA (*P* = 0.014) or SVO (*P* = 0.004) mechanisms. In stroke patients with CE, OD, and UD, glycated albumin levels did not show a statistically significant difference in the presence of END

When comparing the impact of GA on END according to the mechanism of stroke, there was a statistically significant difference in GA values between the END group and non-END group in patients with stroke caused by large artery atherosclerosis (LAA) (*P* = 0.014) or small vessel occlusion (SVO) (*P* = 0.004). In patients with stroke caused by other mechanisms, GA levels did not show a statistically significant association with END (Fig. 1).

In comparing the predictive power of glycemic parameters for END, GA showed a higher AUC value of 0.622 (0.560 to 0.683) on the ROC curve than fasting glucose (0.582) or hemoglobin A1c (0.578) (Figure S1). This difference in predictive power was more evident in the LAA or SVO stroke groups (Figure S2). These results were also consistent with the multivariable logistic regression analysis based on well-known cut-off values (Table S1).

## Discussion

In this study, we demonstrated that higher GA values were closely associated with the occurrence of END in patients with AIS. In particular, GA appeared to be more closely involved in the occurrence of END in patients with LAA or SVO stroke. In addition, GA showed superior predictive power for END occurrence compared with other well-known glycemic parameters.

The exact mechanism underlying the close association between GA values and END is unclear. However, we propose several plausible hypotheses. First, high GA levels may indicate oxidative stress-induced endothelial dysfunction [19]. Albumin accounts for 50–60% of plasma proteins and plays a major role in human antioxidant function [10, 25]. Glycated albumin loses its antioxidant activity, which can increase reactive oxygen species and lead to the breakdown of the blood-brain barrier and damage to endothelial cells [10]. In such an environment, the initial stroke lesion is more likely to increase in size, and larger edema can occur because of impaired clearance through the glymphatic pathway [26–28]. In fact, recent experimental and population studies have reported that glycemic variability, rather than chronic hyperglycemia, is more likely to induce oxidative stress and is closely associated with microvascular complications [11, 15, 21]. In our data, GA values showed a close association with chronic kidney disease, another microvascular disease, while hemoglobin A1c did not show a statistically significant association, supporting this claim. Second, GA can affect atherosclerotic plaques in both cerebral large and small vessels. Similar to other glycemic parameters, GA has been shown to be closely associated with the progression or rupture of atherosclerotic plaques in several studies [10, 13, 17]. The exacerbation of atherosclerotic plaques in the cerebral vessels can contribute to the occurrence of END through in situ thrombosis, artery-to-artery embolism, and branch atheromatous disease [29]. Third, high GA levels may be associated with hemorrhagic complications following thrombolytic therapy. Previous studies have shown a strong association between high GA levels and the occurrence of hemorrhagic transformation following intravenous thrombolysis or intra-arterial thrombectomy [19, 21]. Although our study did not address hemorrhagic transformation, our data also showed a greater difference in GA values between the END and non-END groups in patients who received thrombolytic therapy than those who did not. Last, GA can reduce the effects of anti-platelet agents through platelet aggregation or activation [12, 30]. Therefore, it can lead to an increased risk of END by reducing the acute treatment effects.

There was no significant difference in the GA values according to the AIS mechanism in our study (*P* = 0.189). However, GA showed a better predictive value for END occurrence in patients with LAA or SVO stroke than in those with embolic stroke. Given the previously described mechanisms, such as endothelial dysfunction or plaque instability due to oxidative stress, this may be a natural result. Patients with LAA or SVO stroke can be classified into more detailed subtypes based on their underlying mechanisms (e.g., in situ thrombosis, hypoperfusion, artery-to-artery embolism, lipohyalinosis, and branch atheromatous) [29, 31]. If we conduct a subsequent study using follow-up magnetic resonance imaging in homogeneous patients with LAA or SVO stroke, we expect to identify the exact pathological mechanisms by which GA causes END in patient with AIS.

In our data, GA showed a higher predictive power for END than hemoglobin A1c or fasting glucose. To be more precise, hemoglobin A1c and fasting glucose showed AUC values that could be interpreted as virtually no correlation, and only GA showed a mild level of predictive power for END. As END is a clinical event that occurs during the acute period, it is natural that hemoglobin A1c, which reflects the average glucose concentration over the past 3 months, has poor predictive power [16, 20]. Fasting glucose can be greatly affected by factors such as diet during hospitalization, use of glucose-lowering agents, and accompanying medical conditions, and can temporarily increase by acting as an acute-phase reactant in a phenomenon called “stress hyperglycemia” after stroke occurrence [8, 9]. As various factors are complicatedly involved, fasting glucose’s predictive power for END may be unstable. However, the GA value only needs to be considered for the impact of relatively poor glycemic control. In conclusion, we believe that GA values have a higher predictive power for END than hemoglobin A1c or fasting glucose levels and that the interpretation of the results is simple. Of course, due to the characteristics of GA, it is thought that it will be difficult to monitor and correct it in real time like fasting glucose level. Rather, it would be better to classify high-risk groups for END by measuring GA levels immediately after hospitalization and establish a treatment strategy that involves intensive glucose level monitoring and management in these groups.

There are several limitations to consider when interpreting the results of this study. First, this was a retrospective cross-sectional study. Therefore, we can only present an association between GA values and END; however, this does not imply a causal relationship. By designing a prospective study that rigorously controls additional confounding variables, such as individual patient treatment strategies, comorbidities, and the timing of GA measurements, we may more clearly confirm the causal relationship between GA levels and END. Second, we analyzed the general AIS population. Therefore, to interpret our results, we must comprehensively consider the potential effect of high GA levels on various stroke mechanisms. As previously mentioned, conducting further studies on more specific stroke patient populations, including brain imaging findings (e.g., intracranial/extracranial atherosclerosis, and cerebral small vessel disease), could provide more definitive clues to the pathological mechanisms. Third, we conducted the analysis using only one GA value measured at admission. If we had measured GA values at discharge and analyzed the impact of its changes on END occurrence during the treatment period, we could have explicitly stated the impact of the actual glycemic variability and presented a basis for establishing treatment strategies. Fourth, we presented several theoretically possible mechanisms to explain the association between high GA levels and END. If specific laboratory and radiological factors that may mediate these mechanisms are included as variables or direct biochemical reactions are confirmed through laboratory studies, a clearer pathological mechanism will be defined and targeted therapy will be possible. Last, we used a relatively sensitive definition for END [32]. However, as there was a clear difference in unfavorable outcomes (mRS ≥ 3) at discharge between the non-END group and END group (Figure S3), we believe that our definition of END is clinically acceptable.

## Conclusion

In conclusion, we demonstrated that high GA levels are positively associated with END in patients with AIS. GA can be easily and quickly measured using simple blood tests, and it is inexpensive. As our data demonstrated that GA had a higher predictive value for early outcomes than other glycemic parameters, we anticipate that GA could serve as a good biomarker for the initial evaluation of patients with AIS. Further prospective studies are required to validate our findings.

### Electronic supplementary material

Below is the link to the electronic supplementary material.

Supplementary Material 1

## Data Availability

No datasets were generated or analysed during the current study.

## Abbreviations

GA: Glycated albumin
END: Early neurological deterioration
NIHSS: National Institutes of Health Stroke Scale
TOAST: The Trial of Org 10172 in Acute Stroke Treatment
LDL: Low-density lipoprotein
HDL: High-density lipoprotein
WBC: White blood cell
hs-CRP: High-sensitivity C-reactive protein
mRS: Modified Rankin Scale
ROC: Receiver operating characteristic
AUC: Area under the curve
