# Supplementary material for: High glycated albumin is associated with early neurological deterioration in patients with acute ischemic stroke
Source: BMC Neurol. 2024 Aug 10;24:278. doi: 10.1186/s12883-024-03747-4 (PMC11316286; doi:10.1186/s12883-024-03747-4)
Supplement: Supplementary file 1 — Supplementary Material 1 [file 12883_2024_3747_MOESM1_ESM.docx]

**Figure S1. ROC curves of hemoglobin A1c, glycated albumin, and fasting glucose for the occurrence of END.**


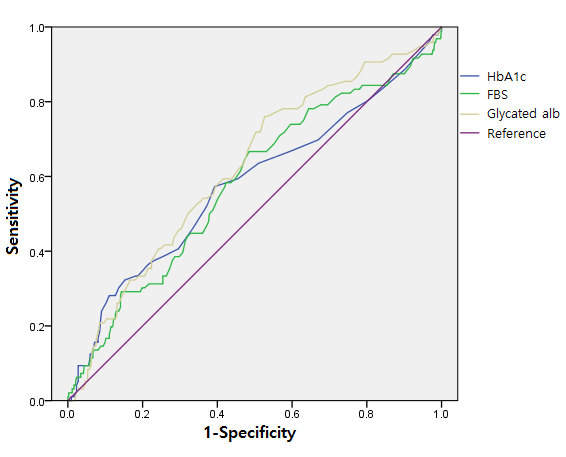


**Figure S2. ROC curves of hemoglobin A1c, glycated albumin, and fasting glucose for the occurrence of END according to the stroke mechanisms.**

**
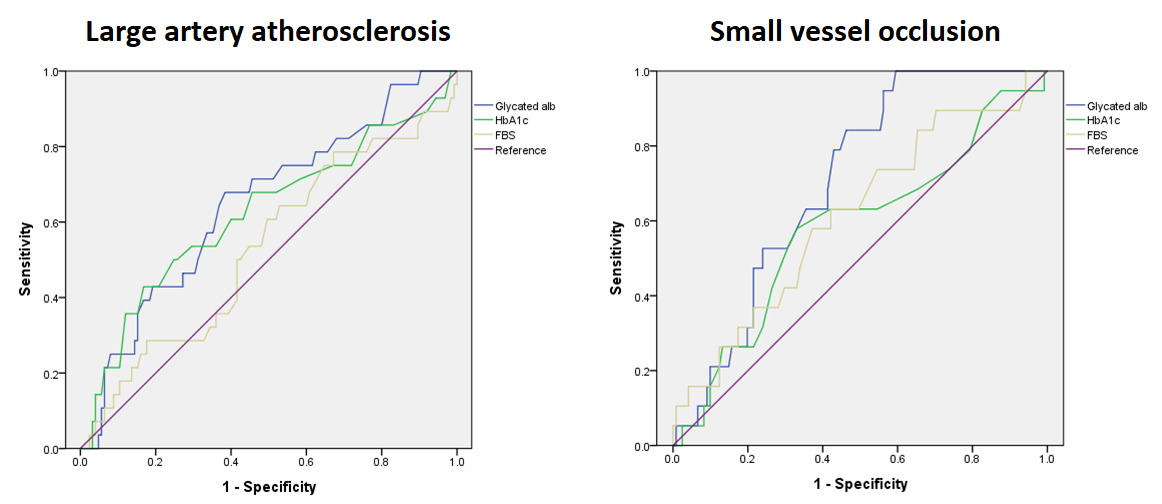
**

**Figure S3. Comparisons of discharge modified Rankin Scale score between groups with and without early neurological deterioration**


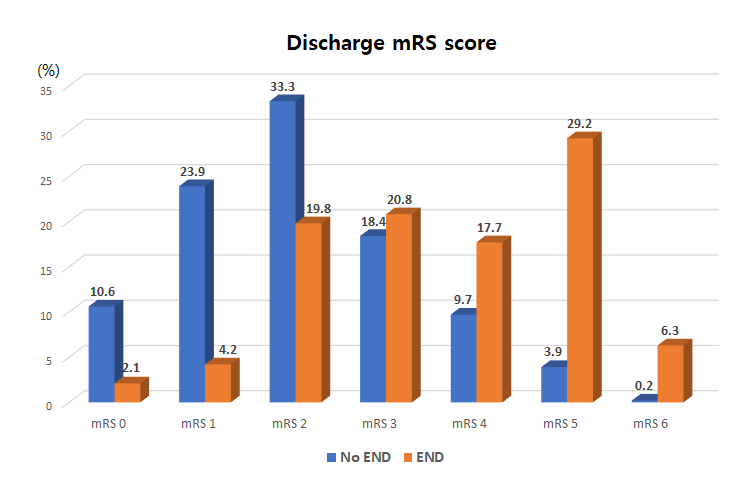


**Table S1. Comparison of odds ratios and 95% confidence intervals in multivariable analysis of various glycemic parameters for END occurrence.**

|  | **Crude OR**  **(95% CI)** | ***P-*value** | **Adjusted OR**  **(95% CI)** | ***P-*value** |
| --- | --- | --- | --- | --- |
| **Model 1 (Glycated albumin)** | | | | |
| Age | 1.02 [1.00-1.03] | 0.094 | 1.01 [0.99-1.03] | 0.256 |
| Diabetes | 1.58 [1.01-2.46] | 0.046 | 1.15 [0.67-1.97] | 0.621 |
| Initial NIHSS score | 1.06 [1.03-1.09] | < 0.001 | 1.04 [1.01-1.08] | 0.021 |
| Thrombolytic therapy | 2.65 [1.54-4.56] | < 0.001 | 2.10 [1.16-3.80] | 0.015 |
| GA > 16.0% | 2.09 [1.34-3.27] | 0.001 | 1.82 [1.05-3.15] | 0.034 |
| WBC counts* | 1.97 [1.02-3.82] | 0.045 | 1.77 [0.89-3.53] | 0.103 |
|  |  |  |  |  |
| **Model 2 (Hemoglobin A1c)** | | | | |
| Age | 1.02 [1.00-1.03] | 0.094 | 1.02 [1.00-1.04] | 0.100 |
| Diabetes | 1.58 [1.01-2.46] | 0.046 | 0.86 [0.38-1.94] | 0.718 |
| Initial NIHSS score | 1.06 [1.03-1.09] | < 0.001 | 1.04 [1.01-1.08] | 0.022 |
| Thrombolytic therapy | 2.65 [1.54-4.56] | < 0.001 | 2.03 [1.12-3.68] | 0.192 |
| Hemoglobin A1c > 6.5% | 2.00 [1.25-3.20] | 0.004 | 2.20 [0.94-5.14] | 0.069 |
| WBC counts* | 1.97 [1.02-3.82] | 0.045 | 1.56 [0.78-3.14] | 0.212 |
|  |  |  |  |  |
| **Model 3 (Fasting glucose)** | | | | |
| Age | 1.02 [1.00-1.03] | 0.094 | 1.01 [1.00-1.03] | 0.128 |
| Diabetes | 1.58 [1.01-2.46] | 0.046 | 1.41 [0.86-2.32] | 0.168 |
| Initial NIHSS score | 1.06 [1.03-1.09] | < 0.001 | 1.04 [1.01-1.08] | 0.015 |
| Thrombolytic therapy | 2.65 [1.54-4.56] | < 0.001 | 1.98 [1.10-3.58] | 0.023 |
| Fasting glucose > 125 mg/dL | 1.52 [0.97-2.37] | 0.065 | 1.28 [0.78-2.11] | 0.327 |
| WBC counts* | 1.97 [1.02-3.82] | 0.045 | 1.61 [0.80-3.23] | 0.181 |

NIHSS = National Institutes of Health Stroke Scale, GA = glycated albumin, WBC = white blood cell

^*^These variables were log-transformed.
